# Fear of falling and postural reactivity in patients with glaucoma

**DOI:** 10.1371/journal.pone.0187220

**Published:** 2017-12-06

**Authors:** Fábio B. Daga, Alberto Diniz-Filho, Erwin R. Boer, Carolina P. B. Gracitelli, Ricardo Y. Abe, Felipe A. Medeiros

**Affiliations:** 1 Duke Eye Center and Department of Ophthalmology, Duke University, Durham, North Carolina, United States of America; 2 Department of Ophthalmology and Vision Science, Federal University of São Paulo, São Paulo, Brazil; 3 Department of Ophthalmology, University of California, San Diego, La Jolla, California, United States of America; Tokai University, JAPAN

## Abstract

**Purpose:**

To investigate the relationship between postural metrics obtained by dynamic visual stimulation in a virtual reality environment and the presence of fear of falling in glaucoma patients.

**Methods:**

This cross-sectional study included 35 glaucoma patients and 26 controls that underwent evaluation of postural balance by a force platform during presentation of static and dynamic visual stimuli with head-mounted goggles (Oculus Rift). In dynamic condition, a peripheral translational stimulus was used to induce vection and assess postural reactivity. Standard deviations of torque moments (SDTM) were calculated as indicative of postural stability. Fear of falling was assessed by a standardized questionnaire. The relationship between a summary score of fear of falling and postural metrics was investigated using linear regression models, adjusting for potentially confounding factors.

**Results:**

Subjects with glaucoma reported greater fear of falling compared to controls (-0.21 vs. 0.27; P = 0.039). In glaucoma patients, postural metrics during dynamic visual stimulus were more associated with fear of falling (R^2^ = 18.8%; P = 0.001) than static (R^2^ = 3.0%; P = 0.005) and dark field (R^2^ = 5.7%; P = 0.007) conditions. In the univariable model, fear of falling was not significantly associated with binocular standard perimetry mean sensitivity (P = 0.855). In the multivariable model, each 1 Nm larger SDTM in anteroposterior direction during dynamic stimulus was associated with a worsening of 0.42 units in the fear of falling questionnaire score (P = 0.001).

**Conclusion:**

In glaucoma patients, postural reactivity to a dynamic visual stimulus using a virtual reality environment was more strongly associated with fear of falling than visual field testing and traditional balance assessment.

## Introduction

Falls are one of the most common and potentially disabling issues among elderly people, jeopardizing their health and independence.[1–7] Vision has an important role in balance control and environment navigation, with previous studies demonstrating that conditions leading to visual impairment are associated to higher risk of falling.[8–11] Glaucoma is a progressive optic neuropathy characterized by degeneration of retinal ganglion cells and their axons and previous studies have reported a higher risk of falling in patients with glaucoma compared to normal subjects.[12–14]

Glaucoma has also been related to greater fear of falling.[15–19] Fear of falling may have major impact in older adults, since it leads to restriction of daily activities,[20] causing reduction in social interaction and mobility,[21,22] loss of confidence,[2,20,21] and depression.[23] In addition, by restricting physical activity, fear of falling may result in further increase in risk of falling. As actual falls may then lead to increased fear of falling, the process can become a vicious circle.[24] Therefore, an assessment of fear of falling is important in order to understand factors associated with fall risk and for development of assistive strategies.

Although standard automatic perimetry (SAP) has been the gold standard test for assessing visual function loss in glaucoma, the association with measures of fear of falling has been inconsistent in the literature.[24] In SAP visual function is measured by assessing sensitivity to detect a static white stimulus against a white background. Due to its simplicity, it is likely that SAP testing does not fully capture certain dynamic aspects of vision that may be important in performing daily activities, such as maintaining balance.

In a previous study, we developed a virtual reality paradigm to evaluate postural control of glaucoma patients in response to dynamic visual stimulation.[11] Postural reactivity was induced by presenting peripheral dynamic visual stimuli in an immersive virtual environment using stereoscopic goggles, while assessing balance through a balance platform. Metrics obtained under this testing paradigm performed significantly better to predict history of falls in patients with glaucoma compared to SAP. However, the relationship between postural reactivity metrics and fear of falling has not yet been investigated. Such relationship could be important in understanding factors that are associated with increased fear of falling in glaucoma.

The purpose of this study was to quantify fear of falling in a cohort of patients with glaucoma and control subjects and investigate the relationship between fear of falling and metrics of postural reactivity obtained using a virtual reality environment.

## Methods

This was a cross-sectional study. Written informed consent was obtained from all participants (including controls and glaucoma patients). Institutional review board approval was obtained and the study was conducted in adherence with the Declaration of Helsinki and to the Health Insurance Portability and Accountability Act.

Participants in this study underwent a comprehensive ophthalmologic examination, and each visited included clinical examination, slit lamp biomicroscopy, visual acuity testing, gonioscopy, dilated fundus examination, intraocular pressure measurement and stereophotography. In addition, participants underwent visual field testing using the Swedish Interactive Thresholding Algorithm Standard 24–2 strategy on a Humphrey Field Analyzer II-i (Carl Zeiss Meditec, Dublin, CA), and they were required to have reliable visual field tests, which was defined as having ≤ 33% fixation losses or false negative errors, or ≤ 15% of false positive errors. Visual acuity was measured using Early Treatment Diabetic Retinopathy Study chart and letter acuity was expressed as the logarithm of minimum angle of resolution. We included only subjects with open angles on gonioscopy. We also investigated about previous systemic diseases (diabetes and hypertension), and also about the systemic use of β-blockers or α-agonists. Subjects were excluded if they presented any other ocular or systemic disease that could affect optic nerve or visual field. Subjects were also excluded if they presented with history of systemic conditions affecting lower limbs, such as arthritis, gout, history of knee or hip replacement, or any other pathology affecting the vestibular system.

Glaucoma was defined by the presence of repeatable abnormal SAP tests (pattern standard deviation with P < 0.05 and/or a Glaucoma Hemifield Test outside normal limits) and corresponding optic nerve damage in at least one eye. Healthy control participants in this study were recruited from the general population through advertisements and were required to be normal on ophthalmological examination and normal appearance of the optic disc on masked grading of stereophotographs. Severity of visual field defect was represented by the integrated binocular mean sensitivity (MS) obtained from monocular SAP 24–2 tests. The integrated binocular MS was calculated as the average of sensitivities of the integrated binocular visual fields obtained according to the binocular summation model described by Nelson-Quigg et al.[25]

All subjects had measurements of weight and height obtained at the time of testing. These were used to calculate body mass index (BMI) for each subject, as the quotient of mass (in kilograms) divided by the square of height (in meters). Level of physical activity was investigated using the Physical Activity Scale for the Elderly (PASE) questionnaire.[26] The scale ranges from 0 to 400, with higher scores indicating greater level of physical activity.[26] History of falls was acquired using the Elderly Fall Screening Test and the Multi-factor Falls Questionnaire.[27]

### Fear of falling evaluation

**Fig 1:**
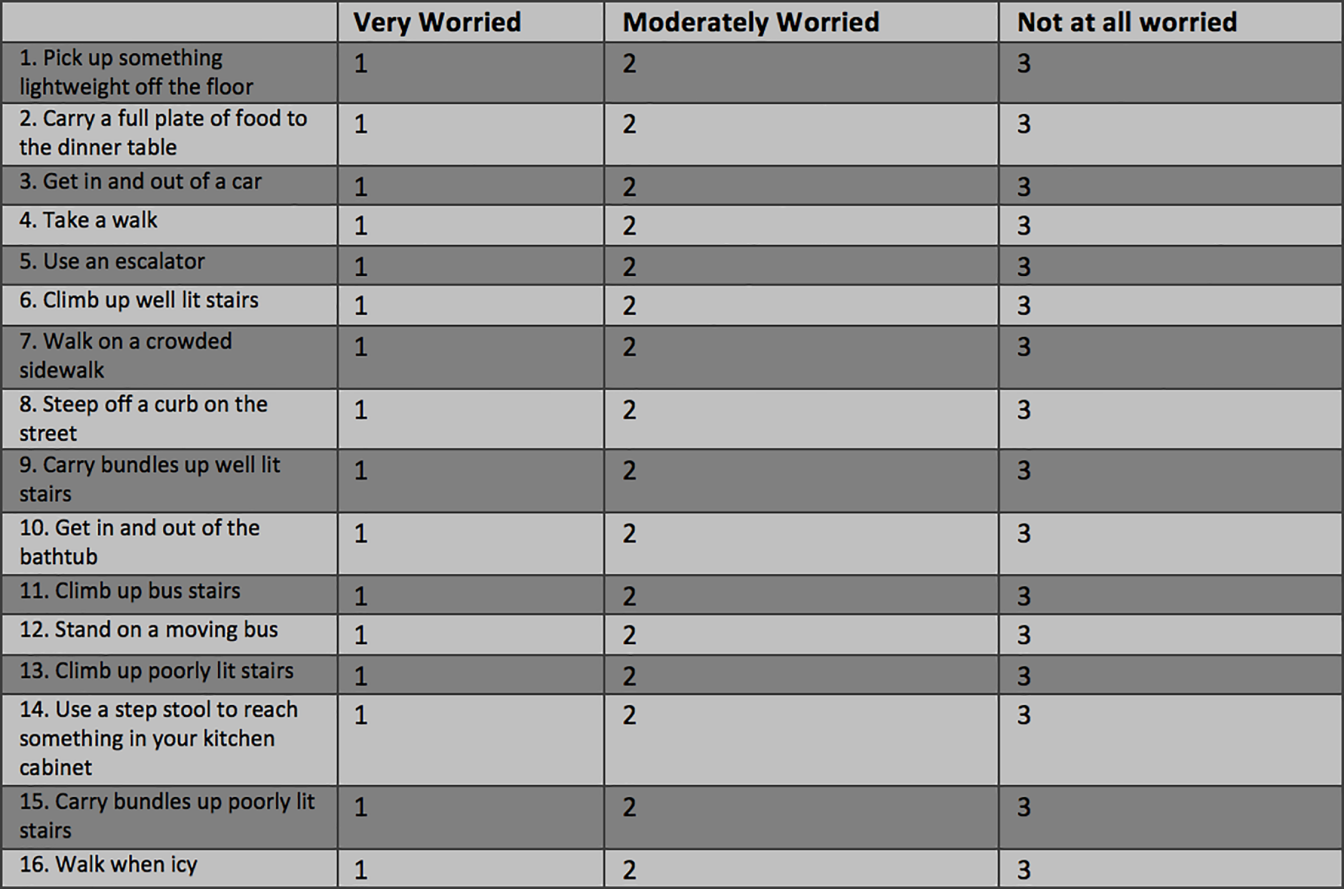
The 16-item University of Illinois at Chicago fear of falling measure scoring questionnaire.

Fear of falling was evaluated using the previously validated 16-item University of Illinois at Chicago fear of falling scoring questionnaire (Fig 1).[28] Questionnaires were administered orally to subjects during an in-person interview. Patients were asked about how much fear they would have if they were to perform any of 16 different tasks, regardless of whether these tasks had been performed recently. Three possible responses were accepted: not at all worried (score 3), moderately worried (score 2), or very worried (score 1).[28] A partial credit item response theory (IRT) model was used to summarize data from the questionnaires and a final score of fear of falling was obtained. By taking into account item difficulty and discrimination, summary scores of fear of falling were obtained for each subject. Scores ranged from -3 to +1, with lower values associated with greater fear of falling.

### Virtual reality environment for assessing postural reactivity

**Fig 2:**
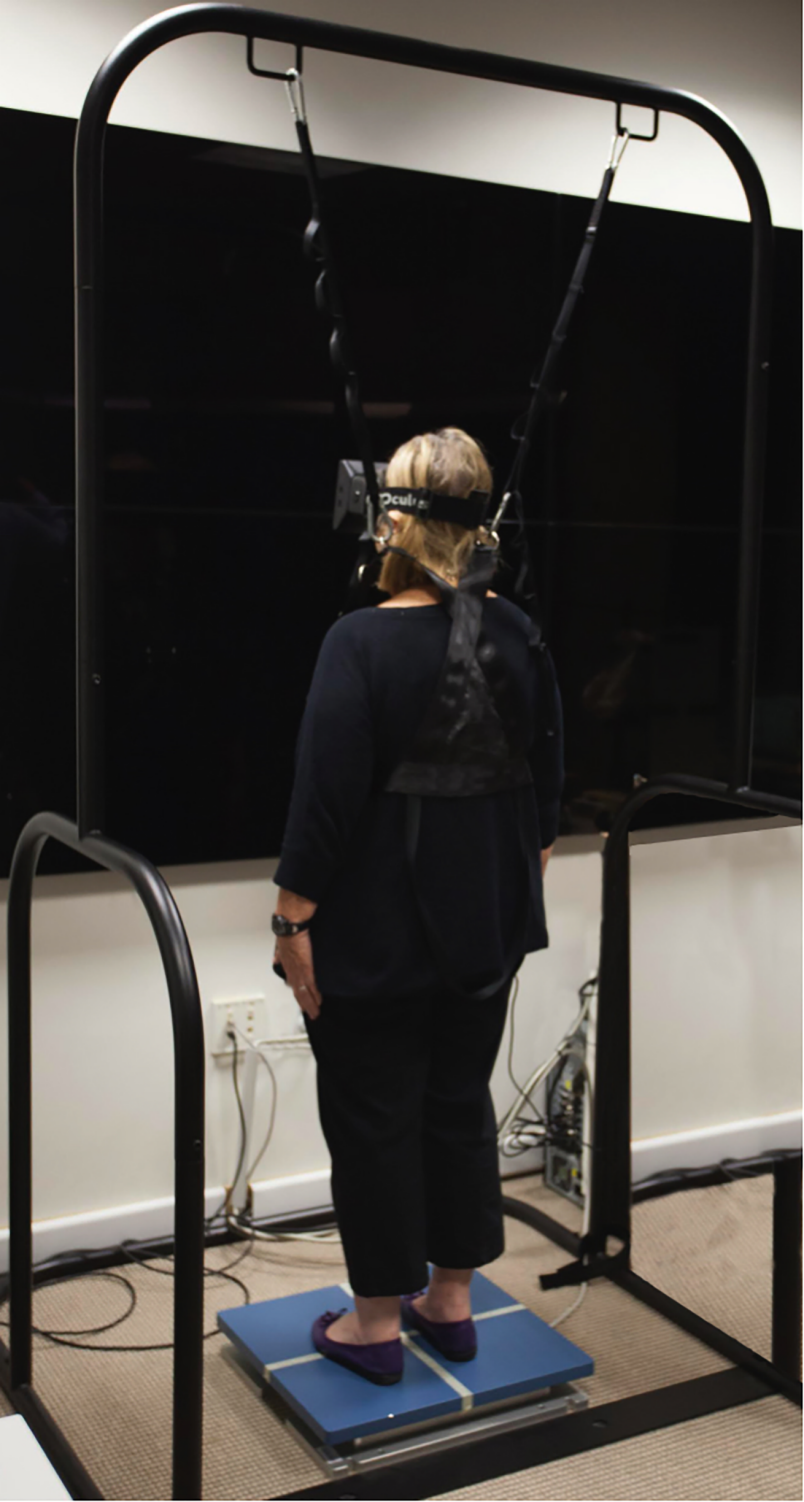
Patient performing the test on the virtual reality environment, while standing on the force platform and wearing the head-mounted goggles.

Postural reactivity to visual information was assessed using an immersive virtual environment with head-mounted stereoscopic goggles (Oculus Rift, Oculus VR, LLC, Irvine, CA) (Fig 2). Details of the testing procedure have been described previously.[11] The Oculus Rift was used to present a stereoscopic 3D image on a binocular field of view of approximately 100 degrees diagonal. Postural stability was evaluated using a force platform (AMTI Optima Human Performance System, Advanced Mechanical Technology, Inc., Watertown, MA). Subjects were supported by a harness system to prevent falling (Handrail and Harness Safety Structure, Bertec Corp., Columbus, OH) (Fig 2). Subjects were required to remove their shoes and stand upright on the center of the force platform with arms by their side and feet close together.

Subjects underwent postural assessment under three conditions:

1. No Oculus Rift (static condition);
2. Oculus Rift in a dark field, without any visual stimulation;
3. Oculus Rift with translational stimulus (dynamic condition).

Postural stability was initially examined without the Oculus Rift. Subjects had both eyes open and were instructed to fixate at a red dot on the wall. Patients were then instructed to put on the Oculus Rift and keep their eyes always opened. Postural stability was then tested with the Oculus Rift showing a completely dark field, i.e., a black screen without any visual stimulation. As no visual input was present, this condition assessed the somatosensory and vestibular contributions to postural control.

In the dynamic condition, postural reactivity was assessed by presenting dynamic visual stimuli in order to induce the sensation of self-motion. This was done by presenting an ecologically valid peripheral background perturbation through the Oculus Rift, a peripheral translational stimulus (tunnel) while the patient fixated down the tunnel. Ecological validity refers to the fact that the visual scene moved as expected when the patients moved his/her head and that the stimulus was what one would visually experience if moving through a tunnel. The peripheral stimulus was presented only in the region outside the central 10 degrees of view, while the patient was instructed to keep fixation on a central red dot.

The translational stimulus was comprised of a sum of four sinusoids with frequencies of 0.1167, 0.2833, 0.5167 and 0.9833 hertz. The amplitude of each sinusoid was inversely related to its frequency to prevent that the highest frequency would dominate. The phases were selected to minimize the difference between maximum magnitude of the combined signal and its root mean square (RMS) magnitude; this process is called cresting and is used to avoid salient signal characteristics caused by an excessively high signal magnitude or an excessively long period of a low signal magnitude. The resulting signal was scaled to yield an amplitude RMS of 0.4 meters and a velocity RMS of 0.5 meters/second for the tunnel stimulus. The benefit of using sums-of-sinusoid stimuli is that the perturbation is unpredictable by the patient unlike the single stimulus. Each test consisted of 15 seconds with a stationary peripheral stimulus followed by 120 seconds of peripheral stimulation. The analysis was performed on the 120 seconds during which the peripheral stimulus was in motion.

For each one of three test conditions described above, torque moments produced in the mediolateral and anteroposterior directions around the center of the force plate were measured. The torque moments are generated when patient’s center of gravity moves causing a change in the center of pressure on the force plate.[29] The standard deviations of the torque moments (SDTM) were calculated as metrics indicative of postural stability and reported in Newton meters (Nm). Standard deviations of the mediolateral and anteroposterior torque moments were computed separately as well as the sum of them. Larger values of SDTM were indicative of worse postural stability.

### Statistical analysis

Descriptive statistics included mean and standard deviation of the variables. Normality assumption was assessed by inspection of histograms and using Shapiro-Wilk test. Fisher’s exact test was used for group comparison for categorical variables. Student *t* test was used for group comparison for normally distributed variables and Wilcoxon rank-sum (Mann-Whitney) test was used for group comparison for continuous non-normally distributed variables.

The association between postural metrics and fear of falling was investigated using linear regression models, where the summary score of fear of falling was used as the dependent variable and the different postural metrics as independent variables. We initially ran univariable models evaluating the association of each variable with the outcome. Subsequently, multivariable models were used adjusting for the potential confounding factors that had a P value < 0.2 in the univariable model. We also investigate the association between severity of visual field defect on SAP and fear of falling.

All statistical analyses were performed using commercially available software Stata, version 14 (StataCorp LP, College Station, TX). The alpha level (type I error) was set at 0.05.

## Results

The study included 35 glaucoma patients and 26 control subjects. Table 1 presents demographic and clinical characteristics of the studied population. There was no significant difference in mean age between the glaucoma and control groups (70.0±11.2 vs. 67.2±11.2 years, respectively; P = 0.262). There were also no statistically significant differences in gender, race, average BMI, PASE scores, binocular visual acuity, and prevalence of hypertension or diabetes between the two groups. As expected, the integrated binocular MS of SAP 24–2 showed worse values in glaucoma patients compared with controls (28.5±3.9 vs. 31.3±1.6 dB, respectively; P<0.001). Subjects with glaucoma reported worse mean scores of fear of falling compared to controls (-0.21 vs. 0.27, respectively; P = 0.039). Diagnosis of glaucoma was also associated with a 2.45 times higher rate of falls in the past 12 months (rate ratio = 2.45; 95% CI: 1.20–4.97; P = 0.013).

**Table 1:** Demographic and clinical characteristics of glaucoma and control subjects included in the study evaluating the relationship between fear of falling and postural reactivity.*.

	Glaucoma (n = 35)	Control (n = 26)	P-value
**Age, years**	70.0 ± 11.2	67.2 ± 11.2	0.262^a^
**Gender, n (%) female**	15 (43)	17 (65)	0.120^b^
**Race, n (%)**			
**White**	18 (51)	15 (58)	0.499^b^
**African American**	11 (31)	10 (39)	
**Asian**	3 (9)	1 (3)	
**Other**	3 (9)	0 (0)	
**Hypertension, n (%)**	22 (68)	15 (68)	0.965^b^
**Diabetes, n (%)**	10 (31)	7 (31)	0.965^b^
**Systemic β-blockers use, n (%)**	18 (50)	7 (25)	
**Systemic α-agonists use, n (%)**	16 (45)	0	
**Score of fear of falling**	-0.21 ± 1.0	0.27 ± 0.7	0.039^a^
**BMI, kg/m**^**2**^	25.0 ± 4.0	25.1 ± 4.8	0.897^c^
**Number of falls in past 12 months**	0.94 ± 1.3	0.4 ± 0.6	0.013^d^
**Summary score of PASE**	156.6 ± 98.5	174.2 ± 96.7	0.526^a^
**MD SAP 24–2 (worse eye), dB**	-5.9 ± 7.8	-0.9 ± 1.9	<0.001^a^
**MD SAP 24–2 (better eye), dB**	-1.95 ± 4.4	0.3 ± 2.3	<0.001^a^
**Binocular MS SAP 24–2, dB**	28.5 ± 3.9	31.3 ± 1.6	<0.001^a^
**Binocular visual acuity, logMAR**	-0.02 ± 0.12	-0.07 ± 0.10	0.074^c^

BMI = body mass index; kg/m^2^ = kilograms per square meter; PASE: Physical Activity Scale for the Elderly; MD = mean deviation; SAP = standard automated perimetry; dB = decibels; MS = mean sensitivity; logMAR = logarithm of the minimum angle of resolution.

*Values are presented as mean ± standard deviation, unless otherwise noted.

^a^ Wilcoxon rank-sum test.

^b^ Fisher’s exact test.

^c^ Student *t* test

^d^ Poisson distribution

Postural metrics during dynamic visual stimulus presentation were significantly associated with fear of falling in glaucoma patients. In the univariable model, each 1 Nm larger SDTM in the anteroposterior direction during dynamic condition was associated with a worsening of 0.32 units in the fear of falling questionnaire score (P = 0.009; R^2^ = 18.8%) (Table 2). SDTM in the anteroposterior direction had a higher association with fear of falling than SDTM in the mediolateral direction (P<0.001). When torque moments in the anteroposterior direction were considered, postural metrics obtained during dynamic visual stimulation were significantly more predictive of fear of falling than those obtained during static (R^2^ = 3.0%; P = 0.005) and dark field conditions (R^2^ = 5.7%; P = 0.007). Fear of falling was not significantly associated with integrated binocular MS (R^2^ = 0.1%; P = 0.855) (Table 2).

**Table 2:** Results of the univariable and multivariable linear regression models for explaining fear of falling in glaucoma patients*.

Characteristic	Univariable Model		Multivariable Model	
	Coefficient (95% CI)	P-value	Coefficient (95% CI)	P-value
**Anteroposterior SDTM in Dynamic, per 1 Nm increase**	-0.32 (-0.55 to -0.08)	0.009	-0.56 (-0.96 to -0.17)	0.007
**Anteroposterior SDTM in Dark Field, per 1 Nm increase**	-0.20 (-0.50 to 0.09)	0.007	0.20 (-0.23 to 0.64)	0.354
**Age, per decade older**	-0.03 (-0.06 to 0.00)	0.059	-0.16 (-0.38 to 0.06)	0.157
**Gender, female**	-0.82 (-1.43 to -0.21)	0.010	-1.04 (-1.61 to -0.47)	0.001
**Race, African American**	0.40 (-0.31 to 1.11)	0.258
**Hypertension**	0.19 (-0.49 to 0.86)	0.576		
**Diabetes**	-0.19 (-0.86 to 0.49)	0.576		
**BMI, per 1 kg/m**^**2**^ **higher** **Number of Falls in past 12 months** **PASE score, per 100 points lower**	-0.03 (-0.12 to 0.05) -0.26 (-0.51 to -0.02) 0.31 (-0.01 to 0.64)	0.454 0.035 0.059	0.03 (-0.19 to 0.24) -0.06 (0.20 to -0.32)	0.818 0.644
**Binocular MS, per 1 dB lower**	0.01 (-0.12 to 0.10)	0.855		
**Binocular visual acuity, per 0.1 logMAR higher**	0.86 (-2.67 to 4.38)	0.621		

CI = confidence interval; SDTM = standard deviations of the torque moments; Nm = Newton meter; BMI = body mass index; kg/m^2^ = kilogram per square meter; PASE = Physical Activity Scale for the Elderly; MS = mean sensitivity; dB = decibel; logMAR = logarithm of the minimum angle of resolution.

*Multivariable model was adjusted for age, gender, number of falls in the past 12 months and PASE score.

**Fig 3:**
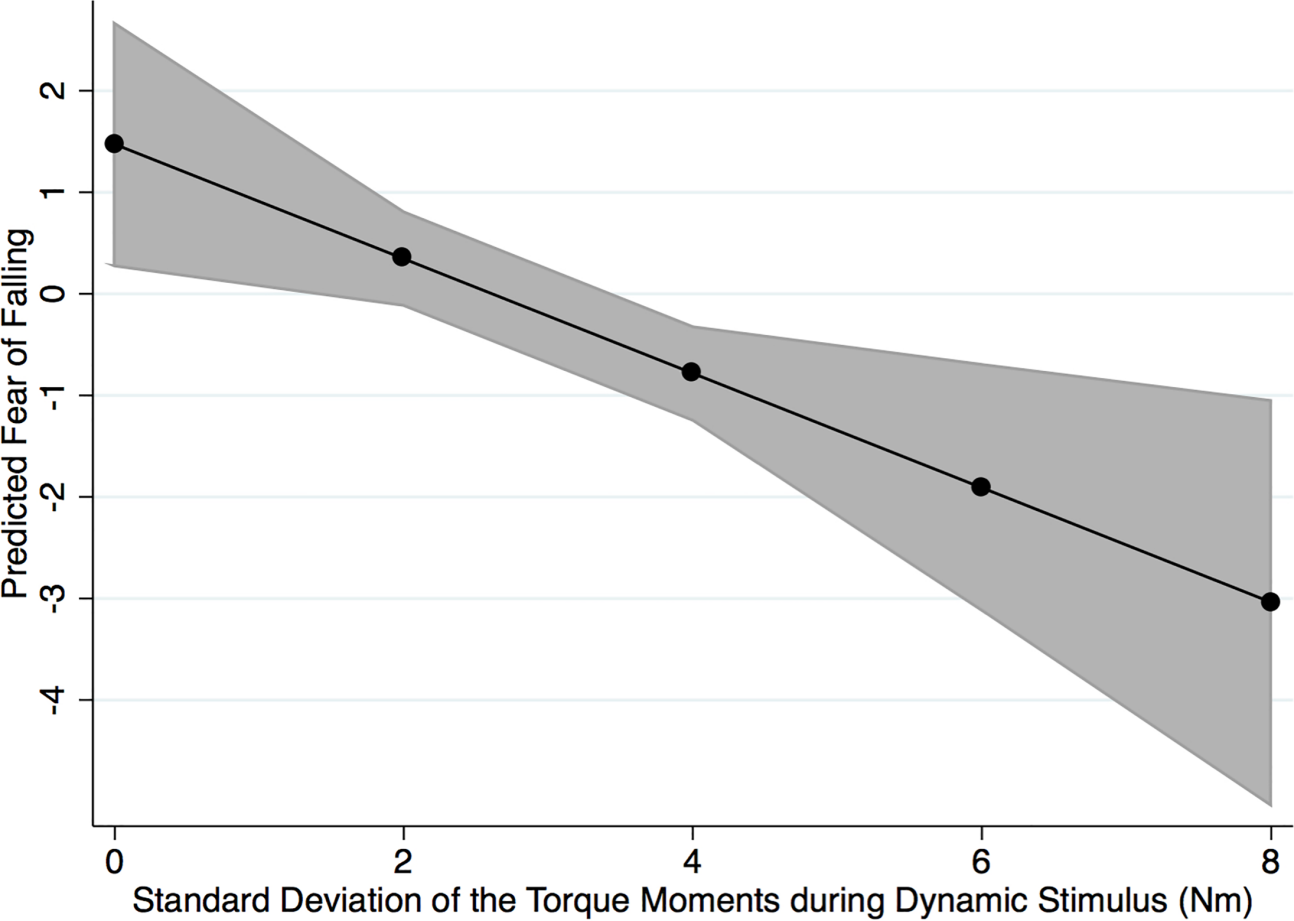
Predicted fear of falling with 95% confidence interval for different values of standard deviation of torque moments (SDTM) in the anteroposterior direction during dynamic visual stimulus presentation. Predicted values were derived from the multivariable model adjusting for confounding factors.

For glaucoma patients, number of falls in the past 12 months was significantly associated with fear of falling (P = 0.035) (Table 2), with a 0.26 worse fear of falling score for each additional fall. Female gender was also associated with 0.82 units worsening in the fear of falling score (P = 0.010) (Table 2). PASE scores were associated with 0.31 units worsening in the fear of falling questionnaire, but results did not reach statistical significance (P = 0.059). In a multivariable model that included age, gender, SDTM in the anteroposterior direction during dark field condition, number of falls in past 12 months, and PASE score, each 1 Nm larger SDTM in the anteroposterior direction during dynamic stimulus was associated with a worsening of 0.56 units in the fear of falling questionnaire score (P = 0.001). The multivariable model had an adjusted R^2^ of 48.8% for predicting fear of falling in glaucoma subjects. (Table 2) (Fig 3). For healthy subjects none of the postural metrics obtained during dynamic condition were significantly associated with fear of falling.

## Discussion

In the present study, we found that metrics of postural reactivity in response to dynamic visual stimulation presented in a virtual reality environment were significantly associated with fear of falling in patients with glaucoma. The postural reactivity metrics showed stronger relationship with fear of falling compared to traditional visual field assessment by SAP. To the best of our knowledge, this is the first study to investigate the relationship between postural reactivity and fear of falling in glaucoma patients. Our findings may help improve the understanding of factors associated with fear of falling in glaucoma and may also potentially assist in the development of management strategies to decrease fear of falling and improve quality of life.

In agreement with previous studies, patients with glaucoma had worse scores on the fear of falling questionnaire compared to control subjects.[15,17] However, lower scores (i.e., worse fear) reported by glaucoma patients were not significantly associated with visual field loss on standard perimetry. Our results contrast to those of Ramulu and colleagues who found a significant association between fear of falling and degree of visual field loss.[15] This could be related to differences in the populations studied. The study by Ramulu and colleagues included mostly patients with moderate and severe visual field loss, with median MD in the better eye of -8dB. In contrast, our study included mostly patients with mild to moderate disease, with median MD in the better eye of only approximately -2dB, although with a wide range from -14.69dB to 2.97dB. Our results indicate that fear of falling in glaucoma patients with relatively mild to moderate disease does not seem to be mediated only by their knowledge of the presence of disease, or by the degree of visual field loss, as indicated by the weak association with SAP results.

The parameter most strongly associated with fear of falling in our study was the SDTM in the anteroposterior direction during dynamic visual stimulus presentation, with each 1 Nm larger SDTM associated with a worsening of 0.32 units in the fear of falling questionnaire score (P = 0.001). SDTM in response to dynamic visual stimuli was more associated with fear of falling than SDTM in the static condition. Previous studies have shown that under static conditions central and peripheral visual fields appear to have equal importance in the control of stance.[30] However, in the presence of dynamic information, peripheral vision plays a crucial role in the control of stance by processing visual information on location and velocity and allowing an adapted postural response to perceived perturbation.[31,32] As glaucoma has a relatively larger effect on peripheral compared to central vision, this may help explain the better performance of postural metrics obtained in dynamic versus static visual stimuli conditions in our study. In fact, previous studies have suggested that differences in postural control may only be detectable when the inducing environment is dynamic, rather than static.[33–35] Our results also indicate that fear of falling in our sample of glaucoma patients does not seem to be mediated by perceived weaknesses in somatosensory and vestibular systems, as indicated by the weaker relationship to SDTM in the dark field condition, i.e., in the absence of any visual stimulation.

We used a virtual reality environment to present dynamic visual stimuli that simulated a tunnel moving in anteroposterior direction (translational stimuli). It is well known that the motion of the environment creates an illusion of self-motion (vection), which in turn induces compensatory postural responses.[36] In a previous study, we have demonstrated that these postural responses are significantly different in glaucoma compared to healthy subjects, with glaucoma patients showing larger SDTM, i.e. greater postural instability in response to the visual stimuli.[11] This increased instability in glaucoma patients may have several explanations. The visual stimuli in our virtual reality paradigm are composed of a sum of sinusoids of different spatial frequencies. In normal subjects, the high spatial frequencies may mask the ability of lower spatial frequencies in effectively inducing vection and, therefore, postural responses would be diminished. For glaucoma patients, it has been shown that retinal ganglion cell loss may result in impaired motion detection, especially for higher spatial frequencies.[37,38] This would then “unmask” the vection-inducing low spatial frequencies, resulting in greater vection and larger postural compensatory responses. Of note, if visual information processing is slow as may happen in glaucoma patients,[39] these compensatory responses may be deficient or inappropriate, leading to postural instability.

It is interesting to note that postural perturbations in the anteroposterior direction (i.e., same direction as of the visual stimuli) were more strongly associated with fear of falling than those in the mediolateral direction. This is in contrast to our previous study investigating the relationship between postural reactivity and risk of falls using the same virtual reality paradigm.[11] In our previous study, we showed that the SDTM in the mediolateral direction was more strongly associated with history of falls than the SDTM in the anteroposterior direction, for the same translational visual stimuli.[11] The higher association with history of falls for the mediolateral SDTM is probably explained by the fact that increased postural perturbations in the direction orthogonal to the visual stimuli may actually be a more important indicator of overall destabilization of the subject and indicate greater risk of falls. However, fear of falling is a subjective perception and, as such, may be more related to the greater sway of the patient that is felt in the anteroposterior direction in response to translational stimuli.

The significant association between fear of falling and our proposed postural metrics could indicate that these metrics might be useful tools to assess the efficacy of interventions designed to reduce fear of falling and risk of falls in glaucoma patients, such as exercise-based interventions. It should be noted, however, that fear of falling in an individual patient may have multiple origins.[40,41] In fact, even the best postural reactivity metric in our study was able to explain only approximately 20% of the variability in fear of falling scores in our sample of glaucoma patients. Other factors were also associated with fear of falling in our study, such as previous history of falls and female gender, whereas age and physical activity were of borderline statistical significance. Importantly, metrics of postural reactivity to dynamic visual stimulation still had significant predictive value even in multivariable models, indicating an independent contribution in explaining fear of falling. The multivariable model was able to explain more than 50% of the variability of fear of falling scores, which could be considered a relatively good predictive ability for such multifactorial and subjective outcome.

Our study had limitations. Due to the cross-sectional design, we were not able to clearly determine the temporal relationship between the proposed postural reactivity metrics and development of fear of falling. In addition, we were not able to investigate the longitudinal relationship between history of falls and fear of falling. However, such relationships are complex and it is likely that simple causal effects cannot be demonstrated. Future longitudinal studies should be able to clarify the role of postural reactivity metrics as proposed in our study and risk of falls and fear of falling in glaucoma. As another limitation of our study, we used only a single questionnaire to investigate and quantify the presence of fear of falling. Although the questionnaire has been previously validated for this purpose, other scales and instruments have also been developed. Future studies should attempt to validate our findings using different instruments to assess fear of falling. As another limitation, it is possible that other unmeasured variables, such as gait abnormalities, use of medications or other systemic conditions, could also be associated with fear of falling in glaucoma and this issue requires further investigations.

In conclusion, evaluation of postural reactivity to a dynamic visual stimulus using a virtual reality environment was more strongly associated with fear of falling in glaucoma patients than visual field testing and traditional balance assessment. These results may contribute to the knowledge of risk factors for fear of falling in glaucoma and may assist in the development of strategies to reduce fear of falling and improve quality of life in patients affected by this condition. In addition, they suggest a promising role for virtual reality in replicating dynamic visual conditions that might be superior to standard perimetry in assessing how vision impacts daily activities in patients with glaucoma.

## Supporting information

S1 Fig Front page of patient from Fig 2 license to publish under the creative commons attribution license. (ZIP)

S2 Fig Back page of patient from Fig 2 license to publish under the creative commons attribution license. (ZIP)

S3 Fig Signed PLOS consent form for publication of Fig 2 in a PLOS journal. (TIF)

S1 Appendix Dataset underlying the findings in the manuscript. (XLSX)
