# Supplementary figures and images for: Fear of falling and postural reactivity in patients with glaucoma
Source: PLoS One. 2017 Dec 6;12(12):e0187220. doi: 10.1371/journal.pone.0187220 (PMC5718417; doi:10.1371/journal.pone.0187220)

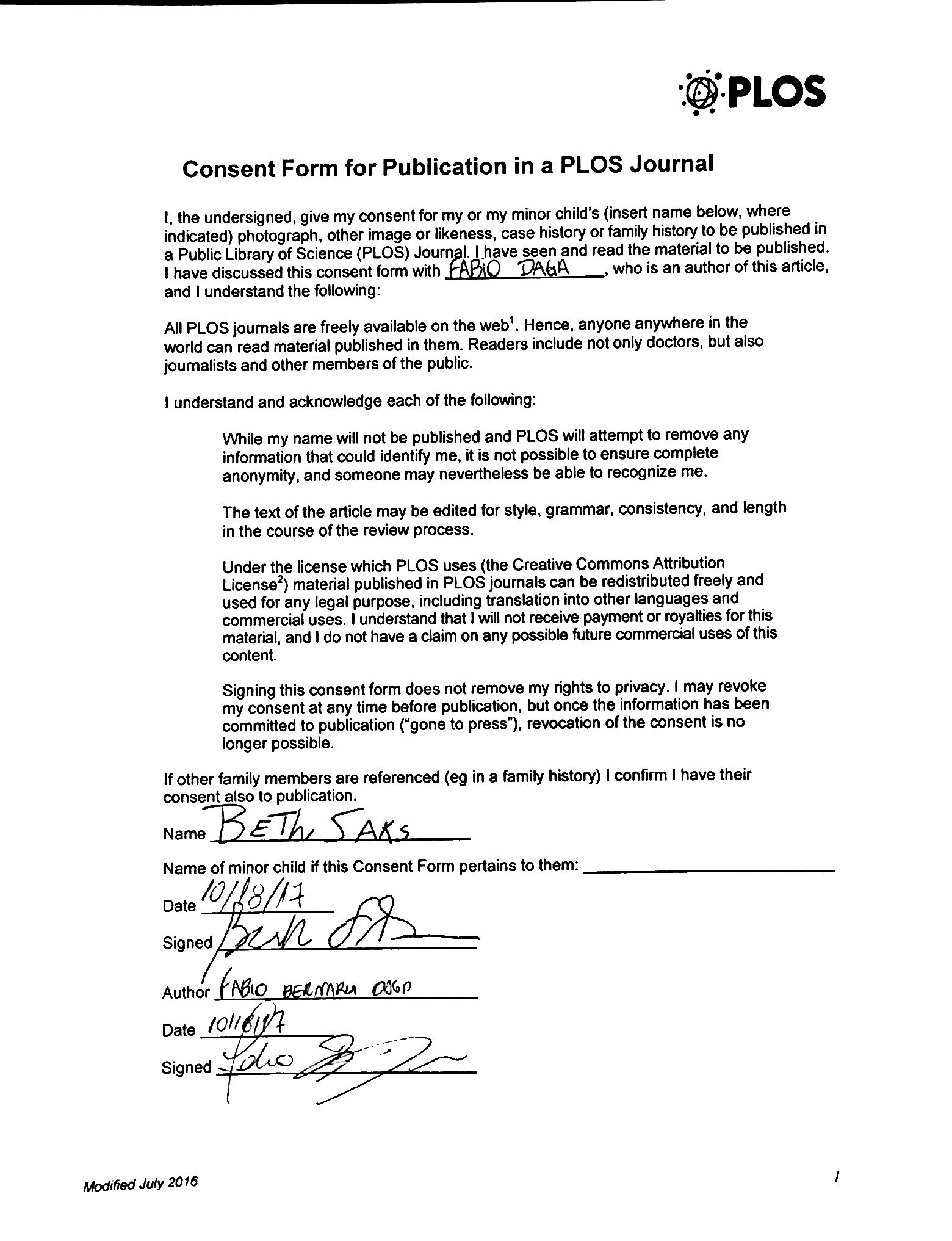

Supplement: S3 Fig — (TIF) [file pone.0187220.s003.tif]
